# Adipose matrix complex: a high-rigidity collagen-rich adipose-derived material for fat grafting

**DOI:** 10.18632/aging.203120

**Published:** 2021-06-09

**Authors:** Ye Li, Pan Zhang, Xue Zhang, Xin Bi, Mengfan Wu, Jialiang Zou, Zijue Wang, Feng Lu, Ziqing Dong, Jianhua Gao

**Affiliations:** 1 Department of Plastic and Cosmetic Surgery, Nanfang Hospital, Southern Medical University, Guangzhou, Guangdong, P.R. China

**Keywords:** fat grafting, stiffness, collagen, rigid supportive filling, mechanical process

## Abstract

Due to the low percentage of collagen, the rigid support capacity of fat grafts remains unsatisfactory for some clinical applications. In this study, we evaluated a strategy in which adipose matrix complex (AMC) was collected via a mechanical process and transplanted for supportive filling of the face. Our AMC samples were collected from adipose tissue by a filter device consisting of a sleeve, three internal sieves, and a filter bag (100 mesh). AMC derived from adipose tissue had fewer cells than Coleman fat, but much higher levels of collagen and stiffness. Retention rates 90 days after transplantation in nude mice were higher for AMC than for Coleman fat (75±7.5% *vs.* 42±13.5%; P < 0.05). In addition, AMC maintained a higher stiffness (~6 kPa *vs.* ~2 kPa; P < 0.01) and stably retained a higher level of collagen. Our findings demonstrate that mechanical collection of AMC from adipose tissue is a practical method for improving fat graft retention and rigid support. This strategy has the potential to improve the quality of lipoaspirates for patients requiring rigid supportive filling.

## INTRODUCTION

Depression or loss of facial tissue is a common problem in plastic surgery [1, 2]. Autologous fat grafting has become an important method for volume augmentation and tissue reconstruction, largely owing to several advantages of adipose tissue: abundance, ease of acquisition, and absence of a graft rejection reaction [3, 4]. However, the rigid support capacity of adipose tissue remains unsatisfactory for some clinical applications, grafting in the nasal base and chin [5, 6].

The stiffness of adipose tissue is 2–4 kPa [7], resulting in unsatisfactory rigid support capacity. The stiffness of adipose depends on the proportion of extracellular matrix (ECM) [8]. The ECM consists of various types of collagen, including collagen I, II, III, IV, and other small-fragment collagens [9, 10]. In particular, collagen I, the most prominent component of ECM distributed throughout the interstitium, constitutes up to 90% of the total connective tissue [11]. Thus, the low proportion of stable ECM and low percentage of collagen I are responsible for the low stiffness of adipose tissue.

Current fat grafting techniques, including centrifugation, mesh filtering, and the Telfa technique do not focus on collagen in adipose tissue [12]. Collagen I can provide mechanical rigidity [13] and also increase stiffness. The higher the proportion of type I collagen, the higher the tissue rigidity [14]. In addition, a high-stiffness environment could stimulate mesenchymal stem cells to secrete more collagen I [15]. Hence, we hypothesized that extraction of the collagen I-rich part of adipose tissue could increase the rigidity of a graft and maintain it for a long time after grafting.

Collagen I is a macromolecular fragment, which fibrillary assemblies could be crowding-enhanced matrix assembly with other type of collagen [16]. Matrix assembly can be easily blocked [17]. Accordingly, the collagen I-rich fraction of adipose tissue could be collected by physical mechanical process.

This study describes a practical strategy for transplantation of the collagen I-rich fraction, adipose matrix complex (AMC), which is obtained by mechanical preparation and purification of ECM from lipoaspirates. In comparison with Coleman fat, AMC contains a higher proportion of type I collagen; therefore, it has a higher stiffness. Consequently, it can be used for clinical filling of areas requiring rigid support (such as the nasal base and chin). We performed measurements to determine the stiffness of, and histological changes in, AMC before and after grafting and compared them with the corresponding properties of Coleman fat.

## RESULTS

### Physical properties of AMC

**Figure 1:**
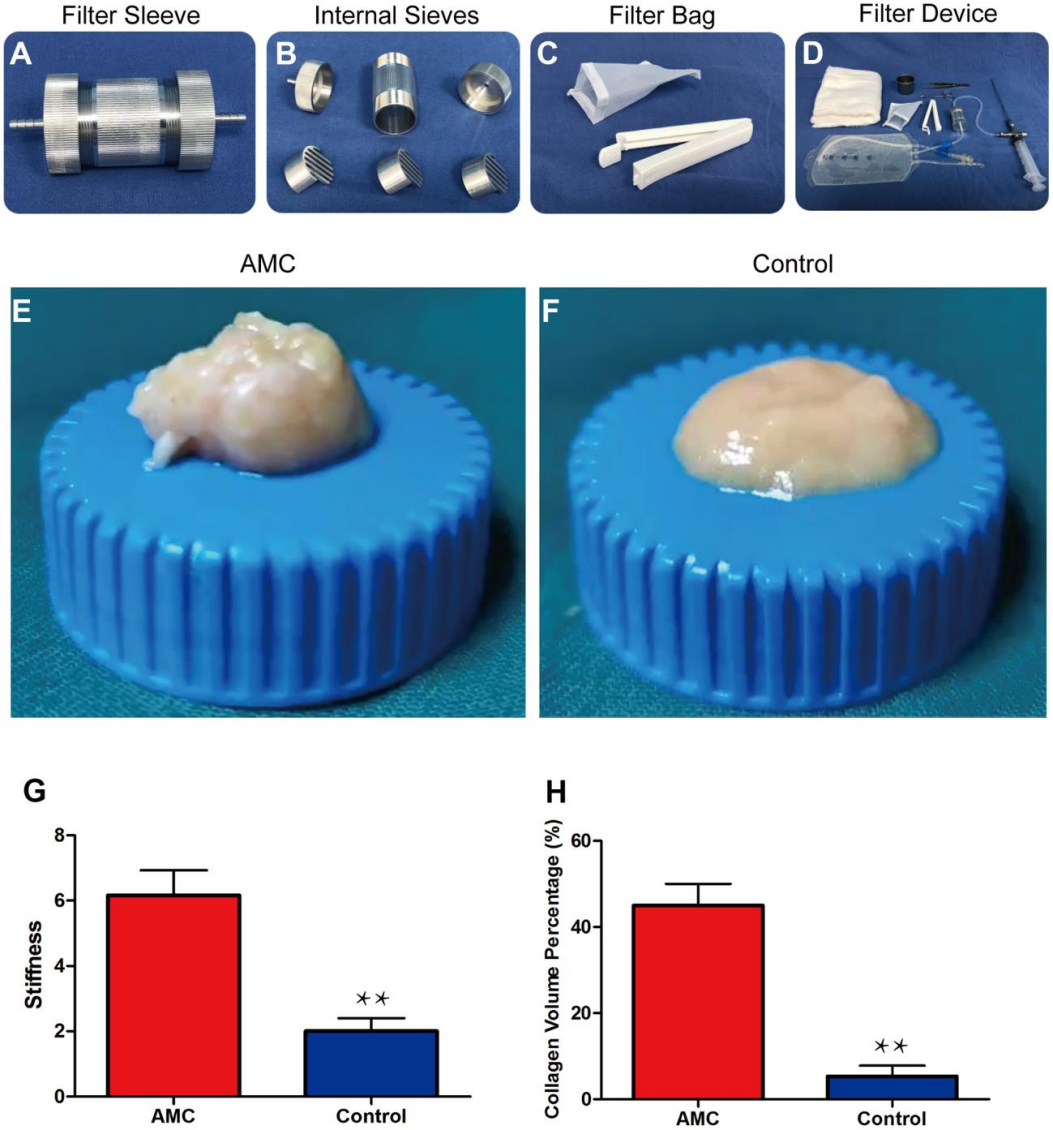
**Preparation and physical properties of AMC.** (**A**) Filter sleeve used to separate and collect AMC. (**B**) Three sieves inside the filter sleeve. (**C**) Filter bag (100 mesh) for dehydrating AMC. (**D**) The entire filter device used to prepare AMC. (**E**, **F**) Appearance of AMC and Coleman fat (control) before grafting. (**G**) Stiffness analysis of AMC and Coleman fat (control) before grafting. (**H**) Collagen volume percentage of AMC and Coleman fat (control) before grafting. Results are presented as the mean ± SD (n = 7 per group). ** control *vs.* AMC; P < 0.01.

General examination revealed that AMC had higher plasticity (Figure 1E, 1F) and higher stiffness than Coleman fat (6.1±0.83 kPa *vs.* 1.9±0.22 kPa, respectively; p<0.01) (Figure 1G), as well as a higher volume percentage of collagen 43±8.5% *vs.* 12±4.5%, respectively; p<0.01) (Figure 1H).

### Collagen and cell percentage of AMC

**Figure 2:**
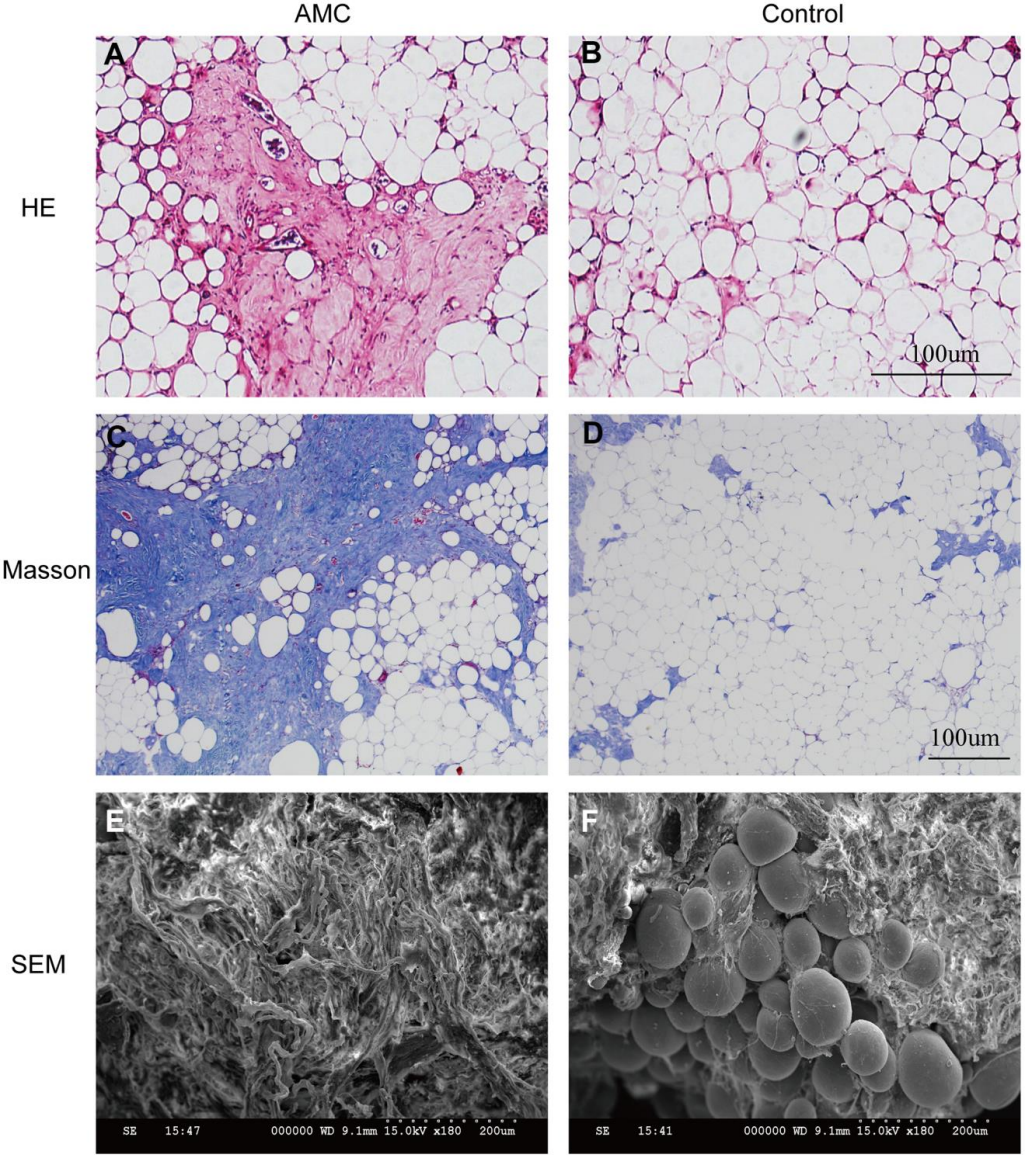
**Histological structure analysis of AMC and Coleman fat.** (**A**, **B**) Hematoxylin/eosin staining of AMC and Coleman fat (control) before grafting. (**C**, **D**) Masson’s trichrome staining of AMC and Coleman fat (control) before grafting. (**E**, **F**) Microstructure of AMC and Coleman fat (control) before grafting, as determined by electron microscopy.

**Figure 3:**
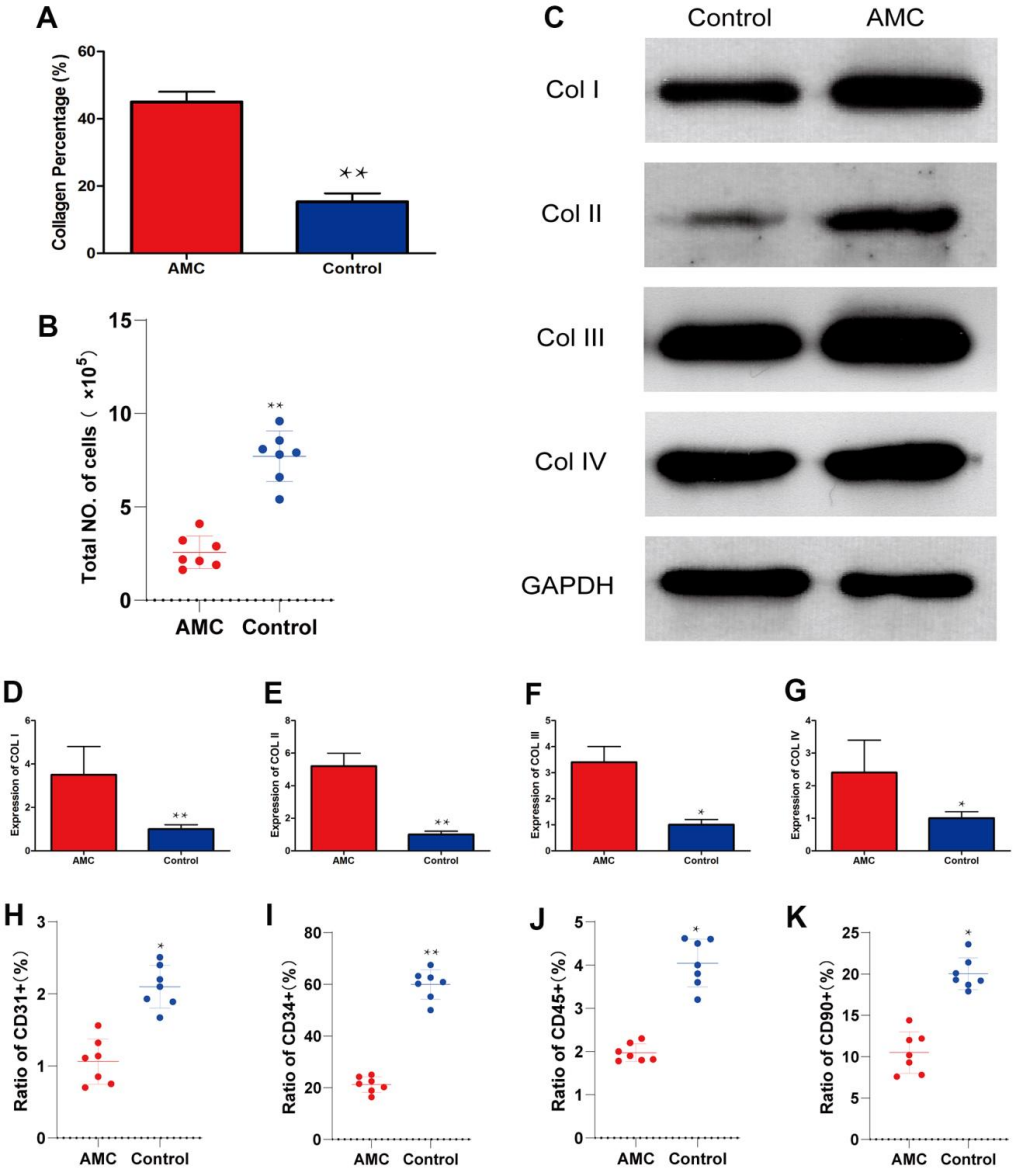
**Collagen and cell percentage in AMC and Coleman fat.** (**A**) Collagen percentage in AMC and Coleman fat (control) before grafting, as calculated by quantification of the positive area in Masson stained sections. (**B**) Total number of cells in AMC and Coleman fat (control) before grafting. (**C**) Expression of Collagen I, II, III, and IV in AMC and Coleman fat (control) before grafting. (**D**) Quantification of collagen I expression in AMC and Coleman fat (control) before grafting. (**E**) Quantification of collagen II expression in AMC and Coleman fat (control) before grafting. (**F**) Quantification of collagen III expression in AMC and Coleman fat (control group) before grafting. (**G**) Quantification of collagen IV expression in AMC and Coleman fat (control group) before grafting. (**H**) Ratio of CD31+ cells in AMC and Coleman fat (control group) before grafting, calculated by flow cytometry. (**I**) Ratio of CD31+ cells in AMC and Coleman fat (control group) before grafting, calculated by flow cytometry. (**J**) Ratio of CD90+ cells in AMC and Coleman fat (control group) before grafting, calculated by flow cytometry. (**K**) Ratio of CD45+ cells in AMC and Coleman fat (control group) before grafting, calculated by flow cytometry. Results are presented as the mean ± SD (n = 7 per group). * control *vs.* AMC; P < 0.05. * * control *vs.* AMC; P < 0.01.

HE and Masson analysis revealed that more ECM was present in AMC than in Coleman fat (Figure 2A–2D). SEM examination confirmed that more fibrous connective tissue was present in AMC (Figure 2E, 2F). Collagen percentage was higher in AMC than Coleman fat (45±3.2% *vs.* 15±3.5%, respectively; p<0.01) (Figure 3A), as was the total number of cells (p<0.01) (Figure 3B). Quantitative analysis revealed that the levels of collagen I (Figure 3D) (p<0.01), collagen II (Figure 3E) (p<0.01), collagen III (Figure 3F) (p<0.05), and collagen IV (Figure 3G) (p<0.05) were higher in AMC than in the Coleman fat.

Almost all cell subsets associated with regeneration, including CD31+ cells (Figure 3H) (p<0.05), CD34+ cells (Figure 3I) (p<0.01), and CD90+ cells (Figure 3J) (p<0.05), were significantly less abundant in AMC than in Coleman fat. The ratio of CD45+ cells was also lower in AMC (p<0.05) (Figure 3K).

### Assessment of grafts

**Figure 4:**
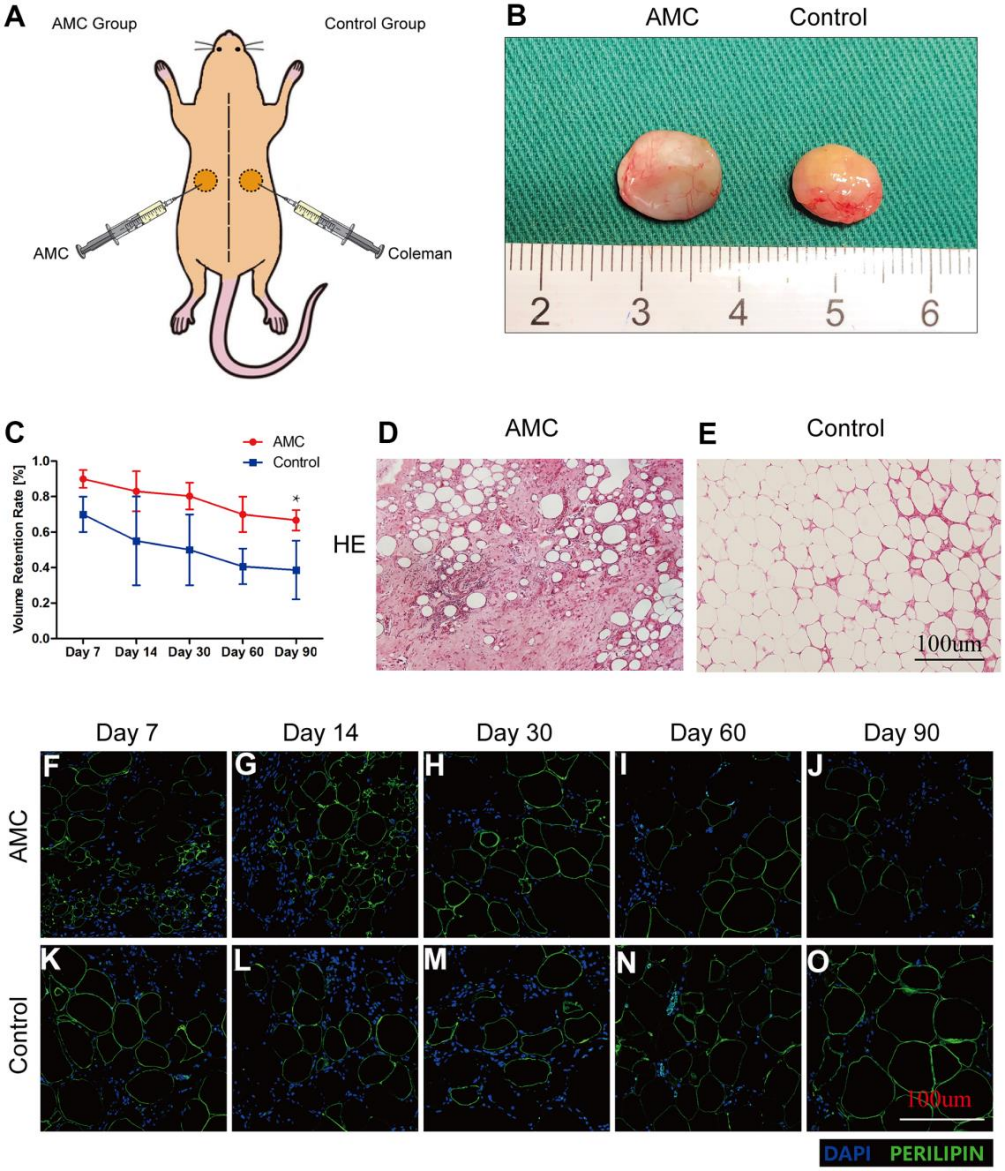
**Retention and tissue structure of AMC and Coleman fat after grafting.** (**A**) Transplantation of AMC and Coleman fat (control group) into nude mice. (**B**) Outward appearance of grafts in AMC and Coleman fat (control group) on Day 90. (**C**) Retention rates of grafts in AMC and Coleman fat (control group) on Days 7, 14, 30, 60, and 90 after grafting. (**D**, **E**) Hematoxylin/eosin staining of grafts in AMC and Coleman fat (control group) on Day 90 after grafting. (**F**–**O**) Immunofluorescence staining of grafts in AMC and Coleman fat (control group) on Days 7, 14, 30, 60, and 90. PERILIPIN+ (green) indicates adipose cells. DAPI+ (blue) indicates cell nuclei. Results are presented as the mean ± SD (n = 7 per group). * P < 0.05, control *vs.* AMC; ** P < 0.01, control *vs.* AMC.

AMC and Coleman fat (Control Group) were grafted onto different sides on the backs of nude mice (Figure 4A). On Day 90, the two types of grafts had a similar outward appearance (Figure 4B). The retention rate of AMC was always higher than that of Control (75±7.5% *vs*. 42±13.5%, respectively). Volume retention decreased slowly in AMC, but decreased rapidly in Control, from Day 7 to 14. Final retention on Day 90 was higher in AMC than in in the Control (Figure 4C).

### Histological structure analysis of grafts in two groups

HE analysis revealed fewer adipocytes in AMC grafts (Figure 4D) than in Control grafts on Day 90 (Figure 4E). Immunofluorescence staining revealed that more cells infiltrated into AMC than Control grafts on Days 7 and 14 (Figure 4F, 4G, 4K, 4L), although the number of infiltrating cells decreased rapidly on Day 30 (Figure 4H, 4M). The area of mature adipocytes was smaller in AMC than Control grafts on Days 60 and 90 (Figure 4I, 4J, 4N, 4O).

### Collagen analysis of grafts in two groups

**Figure 5:**
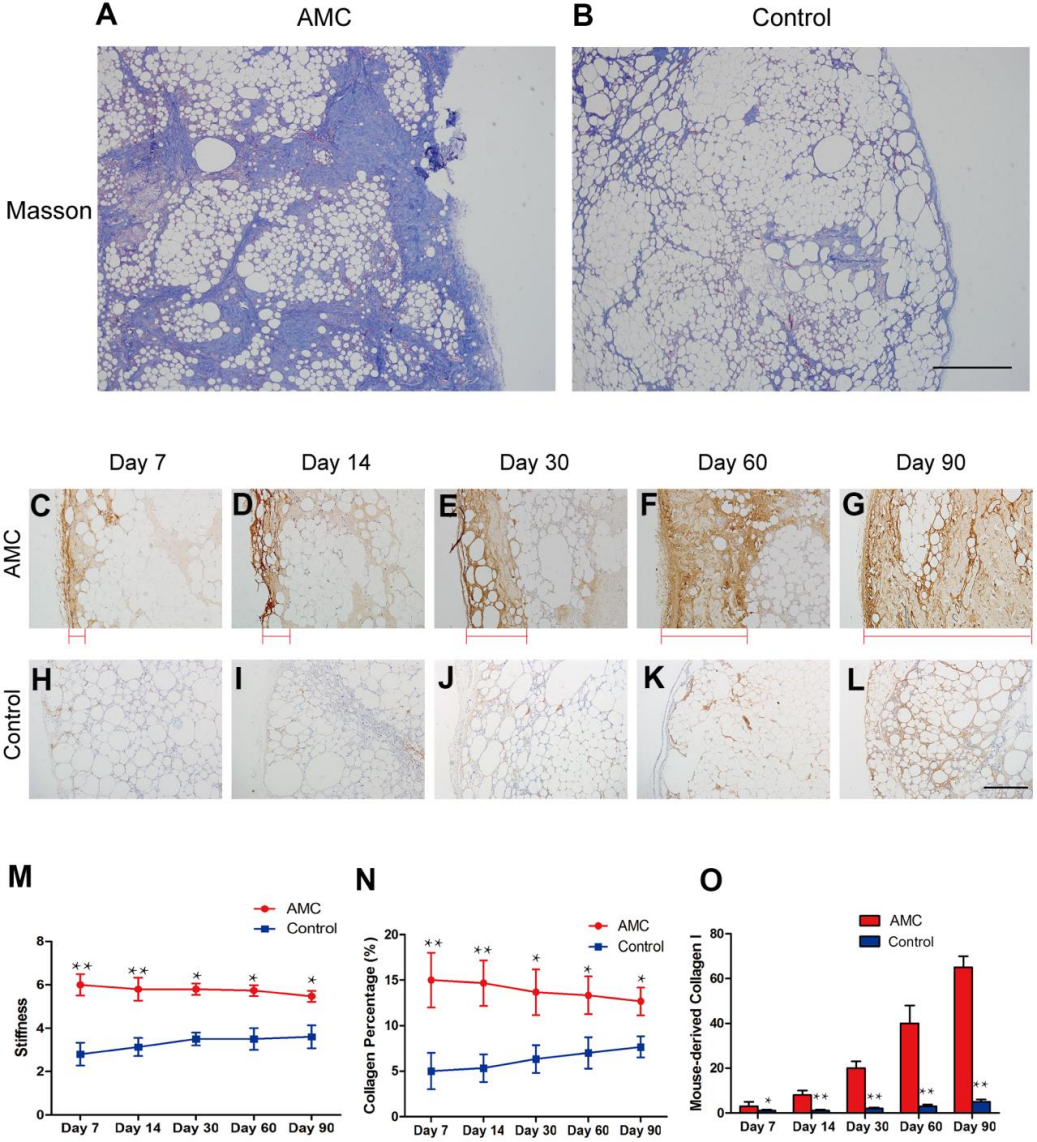
**Changes in collagen in AMC and Coleman fat after grafting.** (**A**, **B**) Masson’s trichrome staining of grafts in AMC and Coleman fat (control) on Day 90 after grafting. (**C**–**L**) Immunohistochemical staining of mouse type I collagen in grafts of AMC and Coleman fat (control) on Days 7, 14, 30, 60 and 90. Red line: type I collagen thickness, from the outermost to the innermost of grafts. (**M**) Stiffness analysis of grafts of AMC and Coleman fat (control) on Days 7, 14, 30, 60, and 90 after grafting. (**N**) Collagen percentage in grafts of AMC and Coleman fat (control) on Days 7, 14, 30, 60, and 90, calculated by quantification of positive area in Masson stained sections. (**O**) Quantification of the area positive for mouse collagen I immunohistochemical staining in grafts of AMC and Coleman fat (control) on Days 7, 14, 30, 60 and 90. Results are presented as the mean ± SD (n = 7 per group). * P < 0.05, control *vs*. AMC; **, control *vs*. AMC; P < 0.01.

Masson analysis revealed much more ECM in AMC (Figure 5A) than in Control grafts (Figure 5B) on Day 90. Immunohistochemistry revealed that in the AMC group, mouse collagen I increased from the outermost to the innermost of the organization (red line) from Day 7 to Day 90 (Figure 5C–5G). By contrast, in Control grafts, mouse collagen I increased steadily (Figure 5H–5L). Mouse collagen I was much more abundant in AMC than Control grafts on Day 90 (Figure 5G, 5L). Accordingly, the stiffness of AMC grafts was higher than that of Control grafts. Additionally, the stiffness of grafts in AMC was maintained at a high level after grafting, similar to the level before grafting (Figure 5M). Quantitative analysis revealed AMC grafts maintained a higher collagen percentage than Control grafts (Figure 5N). In addition, expression of mouse collagen I increased faster and remained at a higher level in AMC than in Control grafts (Figure 5O).

## DISCUSSION

In this study, we investigated a new mechanical strategy for optimizing the components of lipoaspirates for rigid support grafting. We used a filtering device consisting of a sleeve and three internal sieves to collect AMC from adipose tissue. The role of the three internal sieves was to prevent collagen tissue from passing through the sleeve, allowing it to be stored. The role of the filter bag (100 mesh) was to dehydrate collagen tissue. We defined the collagen tissue as AMC. The yield of AMC was about 10%. Using our mechanical process, AMC containing high levels of ECM and collagen I could be collected from Coleman fat. Moreover, AMC had sufficient rigid support capacity. This novel strategy for transplantation of AMC can maintain stable rigid support and improve retention rates.

Traditionally, clinicians use a prosthesis or cartilage to solve the problem of insufficient support [18]. However, for prostheses, immune rejection reaction and absorption have always been problems [19], whereas for cartilage, trauma to the donor site and surgical complications are inevitable [20]. Currently, in the field of tissue engineering, implantation of biodegradable scaffolds in conjunction with fat grafts can be used to improve the rigid support capacity of fat grafting. However, this method has been limited largely to animal experiments due to the need for open surgical incisions, as well as the antigenicity of exogenous scaffold materials [21].

By contrast, AMC can be collected from adipose tissue, which has a high retention rate and causes less trauma and complications. Importantly, AMC also provides sufficient rigid support.

The stable stiffness of tissue depends on the ECM percentage [22]. In this study, we found that the ECM percentage of AMC was greater than 45% before grafting, and that stiffness was about 6 kPa; these values were preserved after grafting. Thus, our results suggest that when the percentage of ECM is greater than 45%, the tissue can provide stable stiffness. Indeed, AMC prepared by our mechanical strategy maintained an ECM percentage above 45%.

AMC contained about 45% collagen by volume, which was much higher than that of Coleman fat (10%). Moreover, expression of collagen I, II, III and IV was higher in AMC. Collagen I, which is the most prominent component of ECM molecules distributed throughout the interstitium, constitutes up to 90% of total connective tissue [11]. In addition, we found that collagen I was the major component in ECM. As we describe, collagen I is macromolecular fragment, which fibrillary assemblies could be crowding-enhanced matrix assembly with other type of collagen [16]. Tissue stiffness increases with the level of collagen I [13, 14]. Thus, the stiffness of AMC, which was capable of providing sufficient rigid support, was much higher than that of Coleman fat.

Additionally, we compared the CD31, CD34, CD90, and CD45 cell ratios between AMC and Coleman fat. CD31 is a marker of vascular endothelial cells [23, 24], which are less abundant in AMC, implying that the angiogenesis capability of AMC might be lower than that of Coleman fat. CD34 is a marker of adipose-derived stem cells (ASCs) [25, 26], and CD45 is considered a marker of immune cells [27, 28], which were also less abundant in AMC, implying that the tissue reorganization capability of AMC might also be lower than that of Coleman fat. More interesting were CD90+ cells, a marker of fibroblasts [29, 30], which were less abundant in AMC than in Coleman fat. Collagen is secreted by various cells [31, 32], including fibroblasts, macrophages, and stem cells [33–35], although fibroblasts are the main cells responsible for collagen secretion [36, 37]. Thus, the collagen regeneration capacity should be lower in AMC than Coleman fat. However, we found that mouse collagen I was increased from the outermost to the innermost of the organization. However, the ECM percentage did not differ among time points after grafting. Together, these observations indicate that the collagen retention mechanism in AMC might involve host-derived collagen regeneration.

The collagen regeneration mechanism appeared to differ between AMC and Coleman fat. After grafting, we observed that more cells infiltrated AMC grafts during the early stage, after which the level of mouse collagen increased. This process deserves our attention. In fact, most cells, including stem cells and endothelial cells, actively migrate from a low-stiffness area to a high-stiffness area [38–40]. Thus, after grafting, the higher stiffness of AMC could result in recruitment of more stromal cells [7, 41], which could explain the faster cell infiltration in the early stage after grafting. In addition, a high level of collagen I can stimulate stem cells to secrete more collagen [42, 43]. This process could maintain a high level of collagen I in tissue after grafting. Also, we found that the collagen percent did not differ among time points after grafting. That is to say, AMC could provide sufficient stiffness, and could maintain stable stiffness after grafting, due to the high level of collagen I in the tissue. We plan to investigate the underlying mechanism in future research.

In the clinic, AMC is collected from adipose tissue after liposuction. Indeed, the mechanical strategy had almost no influence to the adipose tissue. After separation of AMC, adipose tissue could be used for filling, allowing even more smooth injection. Further randomized controlled trials will be required to evaluate the clinical use of AMC for chin and alar base filling, and the efficacy of this strategy should be compared with those of conventional methods.

## MATERIALS AND METHODS

### Fat harvesting and AMC preparation

Human abdominal lipoaspirates were obtained from eight healthy women with no systemic diseases. Liposuction at -0.75 atm of suction pressure was performed with a 3 mm multiport cannula containing several sharp side holes 1 mm in diameter (Tulip Medical Products, San Diego, CA, USA). The fat passed through a filtering device before being collected. The filter device consisted of a sleeve and three internal sieves (Figure 1A, 1B). After filtering, the sieves were removed and the attached tissue was collected. A filter bag (100 mesh) was used for dehydration (Figure 1C, 1D). The resultant tissue was defined as AMC. Finally, the AMC was cut into pieces to ensure that it could pass through the injection needle. [Supplementary Video 1, which shows the whole process of preparing AMC].

For quantification of collagen volume, AMC and Coleman fat were homogenized at 12,000 rpm for 2 min at room temperature using a tissue crushing homogenizer (JJ-2 FK-A, Puyun International Trade Co., Ltd., Shanghai, China). The tissue suspension was centrifuged at 2300 × *g* for 5 min and the upper oil layer was discarded. The isolated collagen at the bottom of the tube was collected and measured.

### Animals

Animals were cared for in accordance with our institutional guidelines. Seventy nude mice of Health SPF level (provided by the experimental animal center, Nanfang Medical University), weighing 15–18 g at age 4–6 weeks (irrespective of sex), were housed in individual cages with a 12 hr light/dark cycle, Animals were provided with standard food and water *ad libitum*.

### Animal model

Mice were anesthetized by intraperitoneal injection of pentobarbital sodium (50 mg/kg). To prepare for grafting, 0.3 ml of prepared AMC or adipose tissue was injected into subcutaneous tissues; this served as the fat graft baseline for each mouse. On Days 7, 14, 30, 60 or 90 post-grafting, the grafts were harvested, carefully separated from surrounding tissue, and their volumes measured. The retention rate of grafts was defined as follows: retention rate (%)=the graft volume at harvest time/injection fat volume (i.e., 0.3 ml)×100%.

### Histological analysis

Samples were fixed in 4% paraformaldehyde, dehydrated, embedded in paraffin, and stained with hematoxylin–eosin and Masson’s trichrome. The samples were then sectioned and examined under an Olympus BX51 microscope. Images were acquired using an Olympus DP71 digital camera.

### Scanning electron microscopy

Fractions were fixed with 2% glutaraldehyde and 1% osmium tetroxide for 1 h. Fat was then dehydrated in acetone, sputtered with gold using an MED 010 coater, and examined under an S-3000N scanning electron microscope (SEM) (Hitachi, Ltd., Tokyo, Japan).

### Stiffness testing

A BOSE ElectroForce load testing device was used to analyze stiffness. The load cell used to measure forces ranged from 0 N to 225 N. The device's electromagnetic actuator allows axial travel of 6 mm. Therefore, a preload was applied to remove slack from tissues and induce elongation. Each tissue sample was clamped, and then a preload (25–30 N) was applied followed by ramp loading. The preload was selected based on preliminary testing. The ramp loading was set to 8 mm/s, and samples were expected to rupture during the application of this loading. The highest force achieved before the sutures tore was electronically recorded for each sample. Finally, the device calculated the tissue stiffness.

### Immunohistochemistry and immunofluorescence

Full-thickness biopsies of the grafts were obtained before and after grafting. Tissue sections were incubated overnight at 4° C with the primary antibody (rabbit anti-mouse type I collagen; 1:200; Sigma) [44]. Tissue sections were washed three times with PBS and then incubated at 37° C for 1 h with biotin-labeled rat anti-rabbit IgG (1:200; Invitrogen) [45]. Signals were observed using the avidin–biotin–horseradish peroxidase detection system. Slides were examined under an Olympus BX51 microscope.

Immunofluorescence staining was performed at 4° C overnight with guinea pig anti-mouse perilipin (1:400; Progen, Heidelberg, Germany) [46]. Tissue sections were washed three times with PBS and then incubated at 37° C for 1 h with rhodamine-conjugated goat anti-guinea pig Alexa Fluor 647 IgG (Abcam, Cambridge, MA, USA) [45]. Nuclei were stained with DAPI (1:200; Sigma). Images were acquired and analyzed under a C1Si confocal laser scanning microscope (Nikon, Tokyo, Japan).

### Western blotting

Total cell lysates were prepared using M-PER Mammalian Protein Extraction Reagent (Thermo Fisher Scientific, Waltham, MA, USA). A BCA protein assay (Thermo Fisher Scientific) was used to estimate the concentration of the protein.

After separation by SDS-PAGE on a NuPAGE electrophoresis system, protein extracts were transferred to immobilon poly-vinylidene difluoride membranes (Millipore, Billerica, MA, USA). Membranes were blocked in 5% milk and immunoblotted with the following primary antibodies: anti-Col I (1:1000; Cell Signaling Technology, Danvers, MA, USA), anti-Col II (1:1000; Cell Signaling Technology), anti-Col III (1:1000; Cell Signaling Technology), and anti-Col IV (1:1000; Cell Signaling Technology). Subsequently, membranes were incubated with the appropriate secondary antibodies. The WesternBreeze Chemiluminescent Detection Kit (ThermoFisher Scientific) was used to detect signals. GAPDH served as an internal control.

### Flow cytometry

Cells were isolated from AMC and Coleman fat at the indicated time points. Briefly, the fat was digested (30 minutes on a shaker at 37° C) in PBS containing 0.075% collagenase. Mature adipocytes and connective tissue were removed by centrifugation at 800 × *g* for 5 minutes. The cell pellets were resuspended and filtered through a 100 μm mesh and a 70 μm mesh. Total cells were then counted. Then, the cells were stimulated for 4 h with 20 ng/ml phorbol myristate acetate (Sigma-Aldrich) and 1 μg/ml ionomycin (Sigma-Aldrich) prior to addition of 10 μg/ml brefeldin A (BFA) (eBiosciences). For the detection of surface markers, cells were stained with CD31 (eBiosciences), CD34 (eBiosciences), CD90 (eBiosciences), and CD45 (eBiosciences) and then incubated for 15 min at 4° C in the dark. After washing, cells were fixed and permeabilized using fixation buffer and permeabilization buffer (BD Biosciences). Acquisition was performed on a Coulter Epics-XL flow cytometer using the System II software (Coulter Corporation, Brea, CA, USA). Data analysis was performed using FCS express software (De Novo Software, Los Angeles, CA, USA).

### Statistical analysis

Mice were grouped according to random numbers generated using SAS 9.4 software (SAS Institute, Inc., Cary, NC, USA). All data were analyzed with IBM SPSS Version 20.0 software (IBM Corp., Armonk, NY, USA). Results are presented as the mean ± standard deviation. Comparisons between multiple time points were assessed by two-way analysis of variance. Comparisons between two groups at a single time point were assessed using an independent Student’s t-test. A two-tailed p-value > 0.05 was considered statistically significant.

## Supplementary Material

Supplementary Video 1
